# Supplementary material for: Gut-derived Flavonifractor species variants are differentially enriched during in vitro incubation with quercetin
Source: PLoS One. 2020 Dec 2;15(12):e0227724. doi: 10.1371/journal.pone.0227724 (PMC7710108; doi:10.1371/journal.pone.0227724)
Supplement: S1 Fig — (DOCX) [file pone.0227724.s001.docx]

**S1 Fig**. **Evolutionary relationships of *F. plautii* strains.**

The evolutionary history was inferred using the UPGMA method. The optimal tree with the sum of branch length = 0.01324911 is shown. The percentage of replicate trees in which the associated taxa clustered together in the bootstrap test (100 replicates) are shown next to the branches. The evolutionary distances were computed using the Maximum Composite Likelihood method and are in the units of the number of base substitutions per site. The scale bar refers to evolutionary distances in substitutions per site. The analysis involved 9 nucleotide sequences: *F. plautii* MC1, *F. plautii* An248*, F. plautii* 1001175st1_C9*, F. plautii* DSM 6740, *F. plautii* 2789STDY5608854, *F. plautii* 2789STDY5834932, *F. plautii* ATCC 29863, *F. plautii* 1_3_50AFAA, and *F. plautii* YL31. All positions containing gaps and missing data were eliminated. There were a total of 1465 positions in the final dataset corresponding to the 16S rRNA gene. Genomes selected for subsequent analyses are labeled with a black circle.
