# Supplementary material for: Gut-derived Flavonifractor species variants are differentially enriched during in vitro incubation with quercetin
Source: PLoS One. 2020 Dec 2;15(12):e0227724. doi: 10.1371/journal.pone.0227724 (PMC7710108; doi:10.1371/journal.pone.0227724)
Supplement: S2 Fig — (DOCX) [file pone.0227724.s002.docx]

**S2 Fig. Evolutionary relationships of *Flavonifractor* sp. strains.**

The evolutionary history was inferred using the UPGMA method. The optimal tree with the sum of branch length = 0.05920312 is shown. The percentage of replicate trees in which the associated taxa clustered together in the bootstrap test (100 replicates) are shown next to the branches. The tree is drawn to scale, with branch lengths in the same units as those of the evolutionary distances used to infer the phylogenetic tree. The scale bar refers to evolutionary distances in substitutions per site. The analysis involved 11 nucleotide sequences: *Flavonifractor* sp. strains An4, An9, An10, An52, An82, An91, An92, An100, An112, An135, An306. All positions containing gaps and missing data were eliminated. There were a total of 230 positions in the final dataset. Genomes selected for subsequent analyses are labeled with a black circle.
