# Supplementary material for: Gut-derived Flavonifractor species variants are differentially enriched during in vitro incubation with quercetin
Source: PLoS One. 2020 Dec 2;15(12):e0227724. doi: 10.1371/journal.pone.0227724 (PMC7710108; doi:10.1371/journal.pone.0227724)
Supplement: S3 Fig — (DOCX) [file pone.0227724.s003.docx]

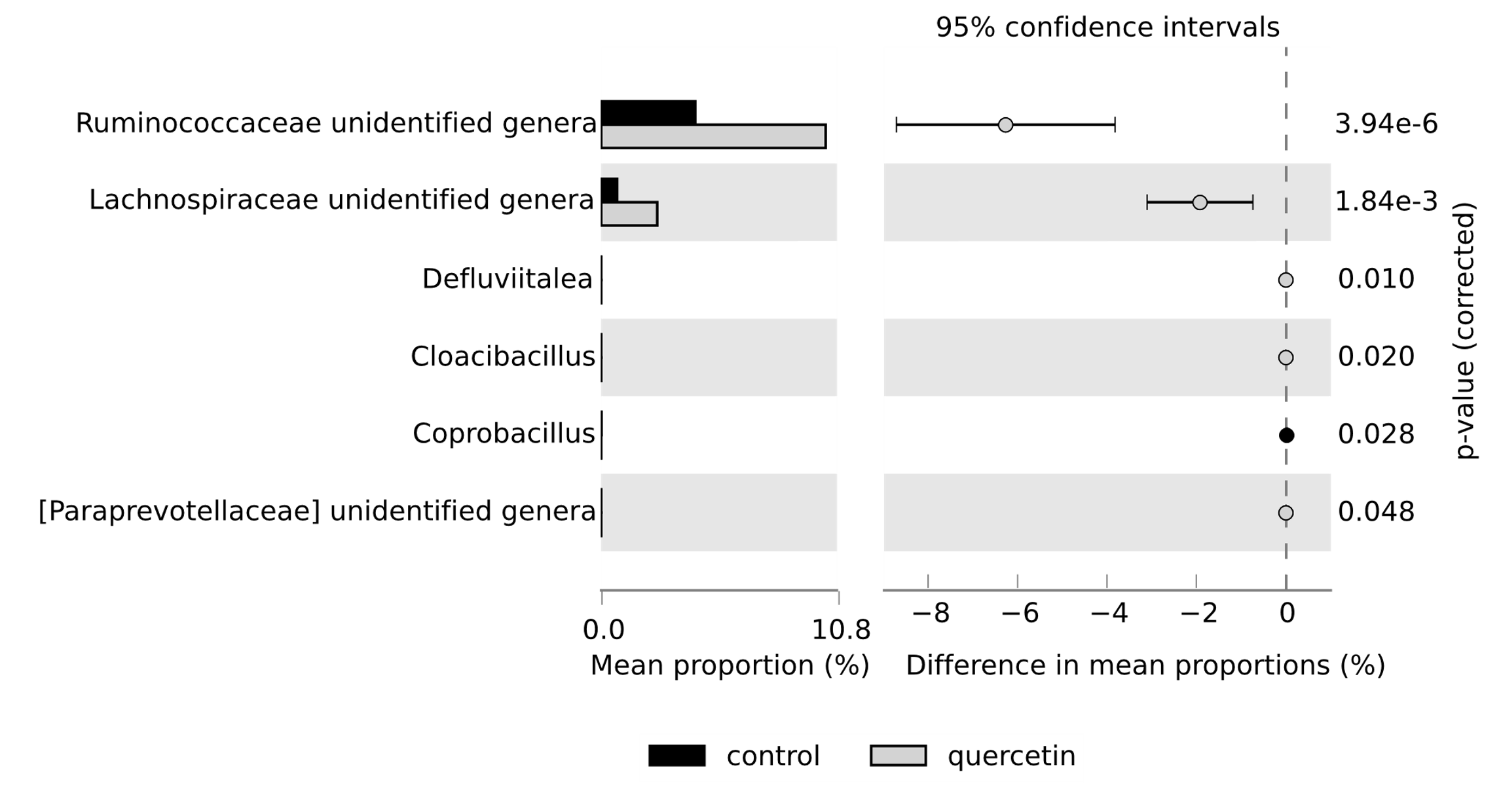


**S3 Fig. Genus abundance profiles from *in vitro* incubations with human fecal samples.**

Extended Error Graph of genera with significant difference between controls (black) and quercetin treatment (gray) calculated with Statistical Analysis of Taxonomic and Functional Profiles (STAMP). Ranking of genera based on p-values. Bacterial taxa whose abundance increased in the presence of quercetin have a negative difference in mean proportion (gray) and those that increased in controls have a positive difference in mean proportion (black).
