# Supplementary material for: Gut-derived Flavonifractor species variants are differentially enriched during in vitro incubation with quercetin
Source: PLoS One. 2020 Dec 2;15(12):e0227724. doi: 10.1371/journal.pone.0227724 (PMC7710108; doi:10.1371/journal.pone.0227724)
Supplement: S4 Fig — (DOCX) [file pone.0227724.s004.docx]

**A B**

**S4 Fig. Proportion of sequences (%) for ASV_65f4 in *in vitro* incubations with human fecal from subject #3.**

STAMP Bar plots for controls at 0 *vs* 72 h of incubation (A) and Quercetin treatment vs controls at 72 h (B).
