# Supplementary material for: Gut-derived Flavonifractor species variants are differentially enriched during in vitro incubation with quercetin
Source: PLoS One. 2020 Dec 2;15(12):e0227724. doi: 10.1371/journal.pone.0227724 (PMC7710108; doi:10.1371/journal.pone.0227724)
Supplement: S5 Fig — (DOCX) [file pone.0227724.s005.docx]

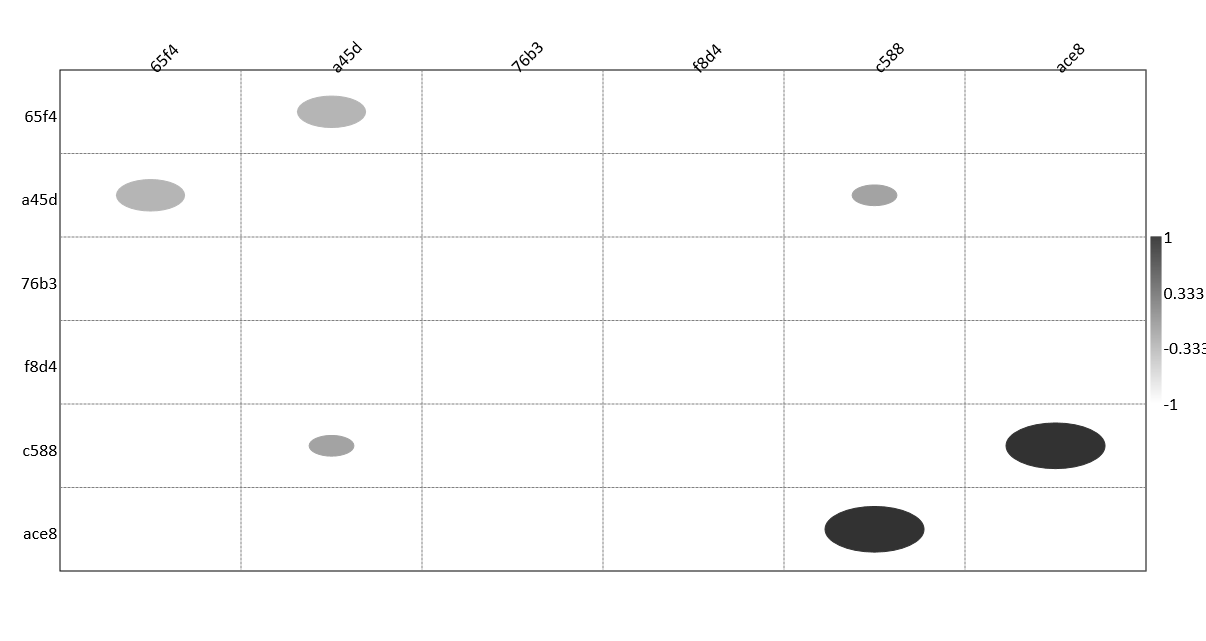


**S5 Fig. Univariate correlations between levels of six fecal taxa (ASVs) enriched in the presence of quercetin *in vitro*.**

Presence of an ellipse represents values below p=0.05. Spearman’s rs and Bonferroni correction were applied. Dark gray is a positive correlation, light gray is a negative correlation. Only treatments with quercetin were analyzed (first and second incubation).
