# Supplementary material for: Gut-derived Flavonifractor species variants are differentially enriched during in vitro incubation with quercetin
Source: PLoS One. 2020 Dec 2;15(12):e0227724. doi: 10.1371/journal.pone.0227724 (PMC7710108; doi:10.1371/journal.pone.0227724)
Supplement: S6 Fig — (DOCX) [file pone.0227724.s006.docx]

**A**


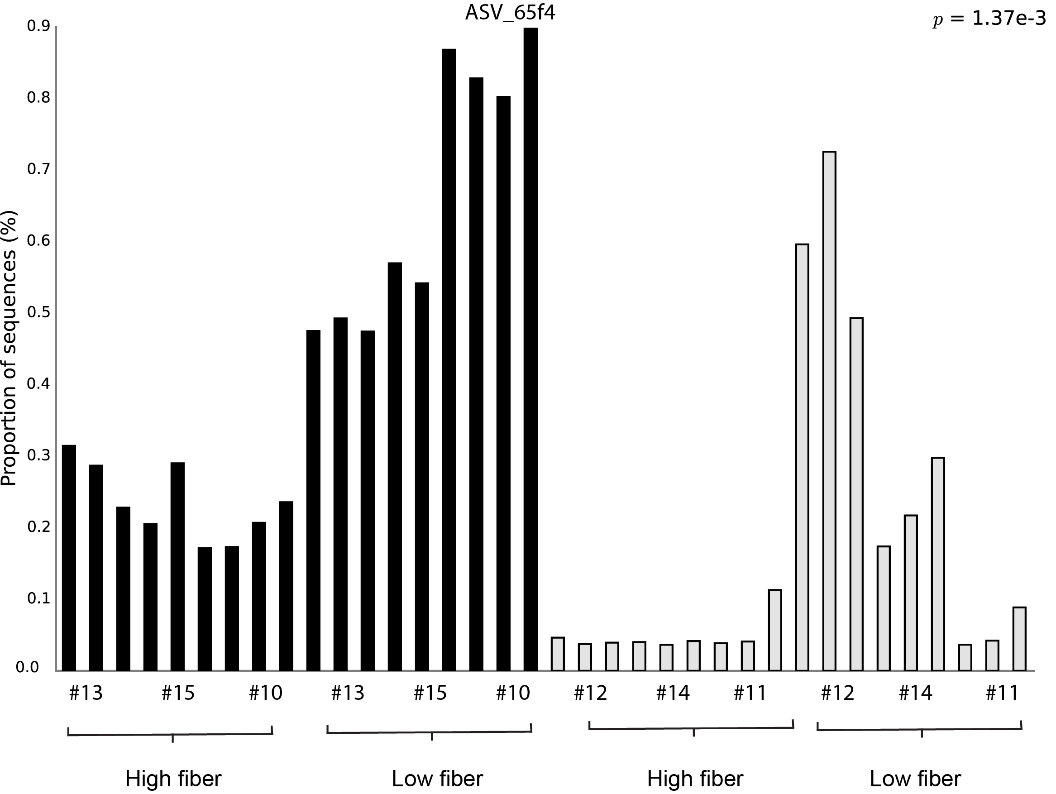


**B**


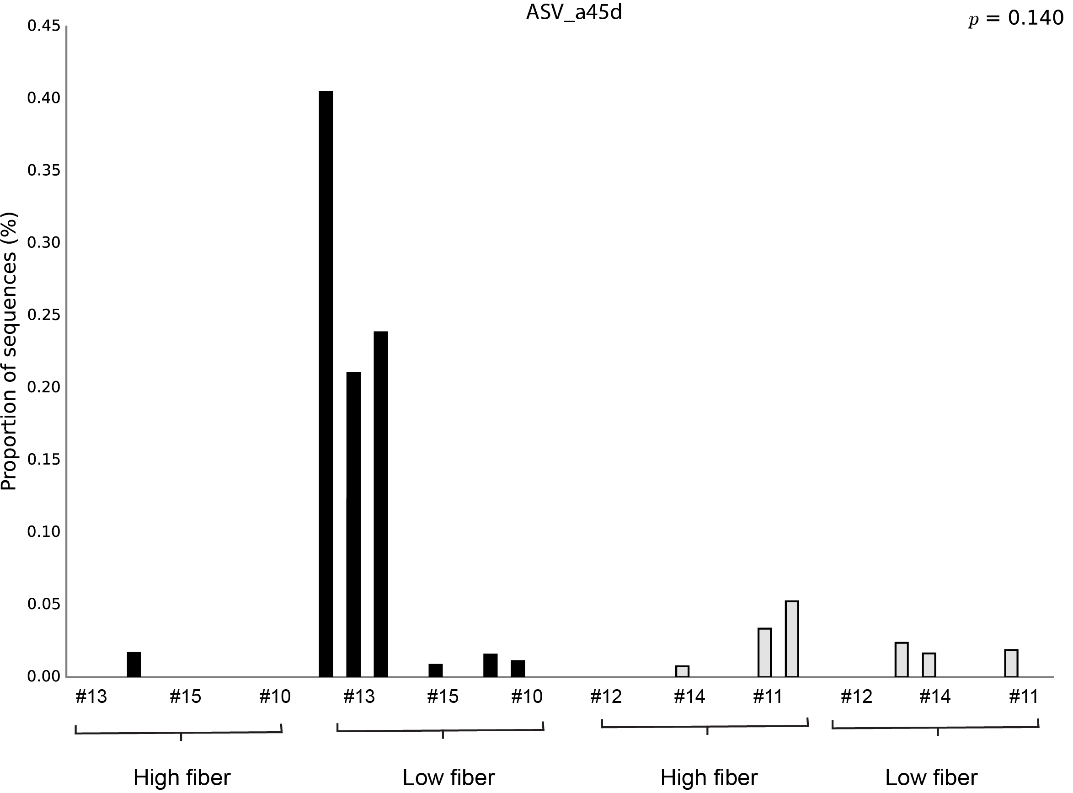


**S6 Fig. Initial relative abundances for ASV_65f4 and ASV_a45d.**

(A) Initial relative abundances for ASV_65f4 in *in vitro* incubations with fecal samples from human microbiota-associated mice (HMAM) (0 days of incubation). (B) Initial relative abundances for ASV_a45d in *in vitro* incubations with fecal samples from human microbiota-associated mice (HMAM) (0 days of incubation). Libraries that were enriched in ASV_65f4 are shown in gray and libraries enriched in ASV_a45d are shown in black.
