# Supplementary material for: Gut-derived Flavonifractor species variants are differentially enriched during in vitro incubation with quercetin
Source: PLoS One. 2020 Dec 2;15(12):e0227724. doi: 10.1371/journal.pone.0227724 (PMC7710108; doi:10.1371/journal.pone.0227724)
Supplement: S7 Fig — (DOCX) [file pone.0227724.s007.docx]

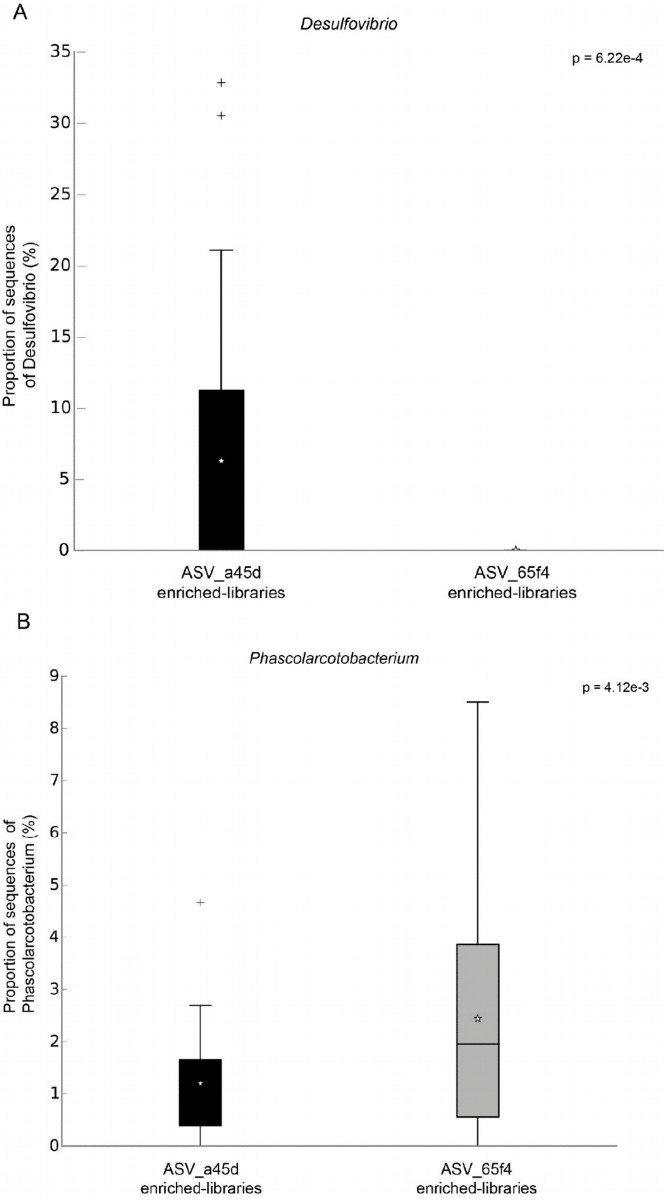


**S7 Fig.** **Box plots for the relative abundances of the genera *Desulfovibrio* and *Phascolarctobacterium.***

(A) Box plots for the relative abundances of the genera *Desulfovibrio* in *in vitro* incubations with quercetin in libraries enriched in ASV_65f4 *vs* libraries enriched in ASV_a45d. (B) Box plots for the relative abundances of the genera *Phascolarctobacterium* in *in vitro* incubations with quercetin in libraries enriched in ASV_65f4 *vs* libraries enriched in ASV_a45d. Analysis for libraries from human subjects #1-#9 grouped by their enrichment in ASV_a45d (black) or ASV_65f4 (gray) is shown. Box plots were calculated with Statistical Analysis of Taxonomic and Functional Profiles (STAMP).
