# Supplementary material for: Gut-derived Flavonifractor species variants are differentially enriched during in vitro incubation with quercetin
Source: PLoS One. 2020 Dec 2;15(12):e0227724. doi: 10.1371/journal.pone.0227724 (PMC7710108; doi:10.1371/journal.pone.0227724)
Supplement: S1 Table — (DOCX) [file pone.0227724.s008.docx]

**S1 Table**. **Genomes used in this study.**

| **Organism name** | **Strain** | **Genome Id** | **Size (bp)** | **Assembly level** | **Genome representation** |
| --- | --- | --- | --- | --- | --- |
| *Flavonifractor plautii*^a^ | YL31 | GCA_001688625.2 | 3,818,478 | Complete Genome | full |
| *Flavonifractor plautii* | 2789STDY5834932 | GCA_001406055.1 | 4,115,051 | Scaffold | full |
| *Flavonifractor plautii* | ATCC 29863 | GCA_000239295.1 | 3,820,124 | Scaffold | full |
| *Flavonifractor plautii* | DSM 6740 | GCA_004345805.1 | 4,431,208 | Scaffold | full |
| *Flavonifractor plautii* | An248 | GCA_002159865.1 | 3,761,516 | Contig | full |
| *Flavonifractor plautii* | MC1 | GCA_901212615.1 | 3,923,577 | Scaffold | full |
| *Flavonifractor plautii* | 1001175st1_C9 | GCA_005844565.1 | 4,011,075 | Scaffold | full |
| *Flavonifractor plautii* | 1_3_50AFAA | GCA_000760655.1 | 4,383,642 | Scaffold | full |
| *Flavonifractor plautii* | 2789STDY5608854 | GCA_001404915.1 | 4,250,184 | Scaffold | full |
| *Flavonifractor*sp. | An4 | GCA_002161245.1 | 3,350,225 | Contig | full |
| *Flavonifractor*sp. | An9 | GCA_002161245.1 | 3,350,225 | Contig | full |
| *Flavonifractor*sp. | An10 | GCA_002161215.1 | 3,882,968 | Contig | full |
| *Flavonifractor*sp. | An52 | GCA_002159385.1 | 2,834,090 | Contig | full |
| *Flavonifractor*sp. | An82 | GCF_002159265.1 | 3,668,665 | Contig | full |
| *Flavonifractor*sp. | An91 | GCA_002159225.1 | 3,603,995 | Contig | full |
| *Flavonifractor*sp. | An92 | GCA_002159175.1 | 3,490,035 | Contig | full |
| *Flavonifractor*sp. | An100 | GCA_002161175.1 | 3,040,137 | Contig | full |
| *Flavonifractor*sp. | An112 | GCA_002161085.1 | 2,958,951 | Contig | full |
| *Flavonifractor*sp. | An135 | GCA_002160795.1 | 3,889,952 | Contig | full |
| *Flavonifractor*sp. | An306 | GCA_002159455.1 | 3,902,886 | Contig | full |

^a^ representative genome
