# Supplementary material for: Gut-derived Flavonifractor species variants are differentially enriched during in vitro incubation with quercetin
Source: PLoS One. 2020 Dec 2;15(12):e0227724. doi: 10.1371/journal.pone.0227724 (PMC7710108; doi:10.1371/journal.pone.0227724)
Supplement: S2 Table — (DOCX) [file pone.0227724.s009.docx]

**S2 Table**. **Average Nucleotide Identity (ANIm) and aligned percentage for 20 genomes belonging to *Flavonifractor* spp.**

|  | **Fp_1_3_50AFAA** | **Fp_2789STDY5834892** | **Fp_2789STDY5834932** | **Fp_1001175st1_C9** | **Fp_An_248** | **Fp_ATCC_29863** | **Fp_DSM_6740** | **Fp_MC1** | **Fp_YL31** | **Fp_An_04** | **Fp_An_09** | **Fp_An_52** | **Fp_An_91** | **Fp_An_112** | **Fp_An_10** | **Fp_An_82** | **Fp_An_92** | **Fp_An_100** | **Fp_An_135** | **Fp_An_306** |
| --- | --- | --- | --- | --- | --- | --- | --- | --- | --- | --- | --- | --- | --- | --- | --- | --- | --- | --- | --- | --- |
| **Fp_1_3_50AFAA** | * | 98.48 [81.09] | 98.57 [76.17] | 98.56 [78.29] | 98.43 [74.34] | 98.49 [75.71] | 98.73 [83.41] | 98.52 [76.06] | 98.36 [75.24] | 85.67 [19.57] | 85.75 [21.08] | 85.81 [19.25] | 85.79 [20.65] | 85.66 [19.94] | 86.78 [38.64] | 85.82 [23.13] | 85.09 [14.23] | 84.21 [5.54] | 85.13 [14.93] | 86.09 [25.45] |
| **Fp_2789STDY5834892** | 98.49 [71.72] | * | 98.34 [68.99] | 98.88 [67.94] | 98.37 [66.71] | 98.47 [64.98] | 98.87 [70.37] | 98.27 [65.16] | 98.32 [62.83] | 85.78 [17.65] | 85.74 [18.42] | 85.86 [17.26] | 86.14 [19.63] | 85.76 [17.71] | 87.25 [35.04] | 86.21 [21.34] | 85.63 [12.36] | 85.30 [5.85] | 85.45 [14.00] | 86.33 [23.12] |
| **Fp_2789STDY5834932** | 98.56 [80.27] | 98.34 [82.31] | * | 98.66 [79.31] | 98.45 [79.26] | 98.63 [77.23] | 98.60 [78.89] | 98.56 [77.97] | 98.48 [78.20] | 85.84 [20.72] | 86.08 [22.15] | 85.80 [20.65] | 86.46 [22.16] | 85.65 [21.03] | 87.07 [41.22] | 86.01 [24.70] | 85.56 [15.82] | 85.24 [5.84] | 86.36 [17.58] | 86.21 [27.55] |
| **Fp_1001175st1_C9** | 98.56 [84.33] | 98.87 [82.89] | 98.66 [81.33] | * | 98.43 [76.63] | 98.67 [82.74] | 98.87 [84.25] | 98.58 [76.83] | 98.40 [77.28] | 85.66 [21.13] | 85.68 [22.50] | 85.73 [21.29] | 85.66 [22.48] | 85.60 [22.22] | 87.02 [41.59] | 86.04 [25.38] | 85.31 [15.87] | 84.72 [6.10] | 85.48 [16.33] | 86.22 [28.26] |
| **Fp_An_248** | 98.44 [84.52] | 98.37 [85.57] | 98.45 [86.14] | 98.46 [81.03] | * | 98.66 [81.58] | 98.57 [83.97] | 98.41 [82.91] | 98.48 [84.75] | 85.67 [22.44] | 85.63 [23.44] | 85.67 [22.62] | 85.66 [23.63] | 85.67 [23.08] | 86.91 [43.72] | 85.82 [26.37] | 85.17 [16.23] | 84.64 [5.73] | 85.28 [17.32] | 86.04 [28.99] |
| **Fp_ATCC_29863** | 98.49 [84.95] | 98.47 [82.90] | 98.63 [82.72] | 98.67 [86.45] | 98.66 [80.44] | * | 98.66 [84.60] | 98.49 [79.64] | 98.39 [80.87] | 85.77 [22.20] | 85.64 [22.65] | 85.70 [22.00] | 86.21 [24.16] | 85.67 [22.97] | 86.87 [42.51] | 85.89 [26.18] | 85.17 [15.95] | 85.65 [6.98] | 86.33 [18.09] | 86.40 [29.42] |
| **Fp_DSM_6740** | 98.72 [82.63] | 98.88 [79.12] | 98.61 [74.51] | 98.87 [77.56] | 98.54 [73.08] | 98.67 [74.46] | * | 98.64 [74.60] | 98.45 [72.09] | 85.64 [19.30] | 85.63 [20.73] | 85.95 [19.58] | 85.65 [20.31] | 85.59 [20.19] | 87.10 [39.70] | 86.15 [23.81] | 85.24 [13.93] | 84.90 [5.58] | 85.35 [14.66] | 86.21 [25.37] |
| **Fp_MC1** | 98.54 [82.46] | 98.27 [80.86] | 98.57 [80.51] | 98.58 [77.26] | 98.42 [79.01] | 98.49 [76.76] | 98.64 [82.53] | * | 98.30 [78.18] | 85.81 [21.26] | 86.08 [22.58] | 85.93 [21.13] | 86.46 [23.50] | 85.66 [21.44] | 86.95 [40.17] | 86.06 [25.19] | 85.16 [15.53] | 84.53 [5.76] | 86.15 [17.31] | 86.33 [28.13] |
| **Fp_YL31** | 98.36 [85.67] | 98.31 [80.93] | 98.48 [84.15] | 98.41 [80.84] | 98.48 [84.49] | 98.38 [81.81] | 98.45 [83.43] | 98.30 [81.16] | * | 85.61 [22.93] | 85.73 [23.76] | 85.78 [22.71] | 85.59 [24.58] | 85.61 [23.42] | 86.84 [42.24] | 85.83 [26.41] | 85.19 [16.10] | 85.06 [6.50] | 85.17 [16.73] | 85.92 [28.41] |
| **Fp_An_04** | 85.68 [26.25] | 85.79 [27.17] | 85.84 [26.48] | 85.66 [26.39] | 85.67 [26.17] | 85.78 [26.56] | 85.65 [26.46] | 85.82 [26.01] | 85.61 [26.69] | * | 97.62 [76.42] | 96.71 [74.06] | 96.90 [78.92] | 96.73 [72.35] | 86.16 [30.30] | 89.36 [60.57] | 85.78 [19.28] | 86.70 [8.39] | 86.34 [21.22] | 86.89 [35.84] |
| **Fp_An_09** | 85.75 [27.16] | 85.75 [27.34] | 86.08 [27.09] | 85.69 [26.86] | 85.63 [26.39] | 85.64 [26.05] | 85.63 [27.31] | 86.09 [26.53] | 85.73 [26.63] | 97.62 [73.05] | * | 96.62 [69.83] | 96.77 [73.90] | 96.79 [70.22] | 87.18 [33.99] | 89.41 [58.77] | 85.98 [20.09] | 87.02 [10.32] | 86.64 [23.24] | 86.68 [35.46] |
| **Fp_An_52** | 85.81 [29.50] | 85.86 [30.22] | 85.79 [30.18] | 85.72 [30.29] | 85.67 [30.20] | 85.70 [29.80] | 85.95 [30.53] | 85.94 [29.43] | 85.77 [30.20] | 96.71 [83.77] | 96.61 [82.66] | * | 97.03 [84.58] | 97.17 [83.09] | 86.17 [34.19] | 89.11 [66.25] | 85.77 [20.85] | 85.52 [9.08] | 85.93 [21.71] | 86.63 [40.38] |
| **Fp_An_91** | 85.79 [25.07] | 86.14 [26.79] | 86.46 [25.57] | 85.66 [25.44] | 85.66 [25.01] | 86.21 [25.77] | 85.65 [24.94] | 86.45 [26.31] | 85.58 [25.63] | 96.90 [69.39] | 96.77 [69.56] | 97.03 [67.00] | * | 97.05 [66.64] | 86.44 [28.26] | 89.12 [54.02] | 85.99 [18.52] | 88.37 [10.64] | 87.44 [21.81] | 87.02 [33.32] |
| **Fp_An_112** | 85.66 [29.19] | 85.76 [29.60] | 85.65 [29.24] | 85.59 [30.13] | 85.67 [29.34] | 85.67 [29.60] | 85.60 [30.03] | 85.66 [28.54] | 85.61 [29.67] | 96.73 [78.20] | 96.78 [79.47] | 97.17 [79.38] | 97.05 [80.33] | * | 86.19 [33.53] | 89.00 [63.70] | 85.83 [21.61] | 86.50 [9.40] | 85.87 [21.97] | 86.84 [40.07] |
| **Fp_An_10** | 86.78 [44.13] | 87.23 [44.90] | 87.05 [43.85] | 87.01 [43.49] | 86.90 [43.30] | 86.86 [42.85] | 87.09 [44.92] | 86.95 [41.70] | 86.83 [42.22] | 86.16 [26.00] | 87.18 [30.75] | 86.17 [25.24] | 86.44 [26.91] | 86.20 [25.94] | * | 87.11 [33.41] | 85.65 [19.85] | 87.36 [9.68] | 85.93 [22.28] | 87.44 [34.34] |
| **Fp_An_82** | 85.83 [27.41] | 86.20 [29.00] | 86.02 [27.63] | 86.04 [27.96] | 85.82 [27.20] | 85.89 [27.47] | 86.16 [28.93] | 86.06 [27.14] | 85.83 [27.24] | 89.36 [53.17] | 89.42 [53.92] | 89.12 [51.27] | 89.11 [52.84] | 89.00 [51.59] | 87.11 [34.80] | * | 86.57 [20.72] | 87.70 [10.26] | 87.41 [23.83] | 87.13 [37.45] |
| **Fp_An_92** | 85.09 [17.97] | 85.62 [17.71] | 85.54 [18.53] | 85.31 [17.95] | 85.17 [17.74] | 85.17 [17.59] | 85.23 [17.92] | 85.16 [17.81] | 85.19 [17.54] | 85.76 [17.95] | 85.98 [19.73] | 85.75 [16.91] | 85.97 [19.23] | 85.81 [18.28] | 85.65 [21.98] | 86.57 [21.80] | * | 86.03 [9.06] | 98.91 [93.42] | 86.14 [20.20] |
| **Fp_An_100** | 84.23 [7.97] | 85.28 [9.44] | 85.26 [7.79] | 84.73 [8.23] | 84.66 [7.12] | 85.67 [8.65] | 84.91 [8.16] | 84.55 [7.52] | 85.08 [7.60] | 86.70 [8.72] | 87.02 [11.49] | 85.52 [8.20] | 88.37 [12.40] | 86.51 [9.09] | 87.36 [11.74] | 87.70 [12.31] | 86.03 [10.24] | * | 87.39 [13.13] | 87.85 [12.37] |
| **Fp_An_135** | 85.13 [16.58] | 85.45 [17.79] | 86.35 [18.48] | 85.48 [16.63] | 85.28 [16.85] | 86.33 [17.86] | 85.36 [16.73] | 86.14 [17.73] | 85.16 [16.29] | 86.33 [17.61] | 86.64 [20.36] | 85.91 [15.97] | 87.43 [20.03] | 85.86 [16.88] | 85.93 [21.71] | 87.40 [22.40] | 98.91 [84.35] | 87.39 [10.71] | * | 86.79 [20.63] |
| **Fp_An_306** | 86.08 [29.02] | 86.32 [30.13] | 86.20 [29.07] | 86.20 [29.52] | 86.03 [28.40] | 86.39 [29.60] | 86.20 [29.23] | 86.32 [29.32] | 85.91 [27.71] | 86.91 [29.80] | 86.69 [30.52] | 86.66 [29.21] | 87.04 [31.18] | 86.84 [30.40] | 87.45 [33.66] | 87.13 [35.29] | 86.14 [18.51] | 87.83 [10.12] | 86.79 [21.67] | * |

First number, average nucleotide identity percentage (with two decimals)

Second number in brackets, coverage percentage (with two decimals).
