# Supplementary material for: Gut-derived Flavonifractor species variants are differentially enriched during in vitro incubation with quercetin
Source: PLoS One. 2020 Dec 2;15(12):e0227724. doi: 10.1371/journal.pone.0227724 (PMC7710108; doi:10.1371/journal.pone.0227724)
Supplement: S3 Table — (DOCX) [file pone.0227724.s010.docx]

**S3 Table. Concentration (mM) in culture of Quercetin and metabolites.** Quercetin and metabolites related to flavonoid degradation for *in vitro* incubations with human fecal samples.

| Subject | DOPAC | PCA | C | E | H | I + M | K | Quercetin |
| --- | --- | --- | --- | --- | --- | --- | --- | --- |
| #1 | **0.362** | <0.021 | 0.015 | 0.067 | <0.004 | <0.021 | 0.024 | <0.004 |
| #2 | **0.385** | 0.025 | <0.004 | <0.004 | <0.004 | <0.021 | <0.021 | <0.004 |
| #3 | **0.351** | 0.025 | <0.004 | 0.006 | 0.009 | <0.021 | <0.021 | <0.004 |
| #4 | **0.385** | 0.027 | <0.004 | <0.004 | <0.004 | <0.021 | <0.021 | <0.004 |
| #5 | **0.356** | 0.024 | <0.004 | 0.008 | <0.004 | <0.021 | <0.021 | <0.004 |
| #6 | **0.399** | 0.025 | <0.004 | <0.004 | <0.004 | <0.021 | <0.021 | 0.005 |
| #8 | **0.377** | <0.021 | 0.036 | 0.004 | 0.005 | <0.021 | <0.021 | <0.004 |
| #9 | **0.325** | 0.022 | 0.005 | 0.006 | 0.008 | <0.021 | <0.021 | <0.004 |

3,4-dihydroxyphenylacetic acid (DOPAC)**,** 3,4-dihydroxybenzoic acid (PCA); 3,4-dihydroxyphenylpropionic acid (C); 3-hydroxybenzoic acid (E); 3-hydroxyphenylacetic acid (H); 3-(3-hydroxyphenyl) propionic acid (M) + phenylacetic acid (I); benzoic acid (K). A number after the symbol “<” indicates less than the minimum detectable value. Results from 2 replicates. Sample #7 was not measured. Samples were measured after completion of quercetin degradation (72 h).
