# Supplementary material for: Gut-derived Flavonifractor species variants are differentially enriched during in vitro incubation with quercetin
Source: PLoS One. 2020 Dec 2;15(12):e0227724. doi: 10.1371/journal.pone.0227724 (PMC7710108; doi:10.1371/journal.pone.0227724)
Supplement: S4 Table — (DOCX) [file pone.0227724.s011.docx]

**S4 Table.** **Statistical significance of Amplicon Sequence Variants (ASVs) enriched in quercetin treatments**. ASVs significantly enriched in quercetin treatments *vs.* controls derived from *in vitro* incubations with human fecal samples.

|  | **Human subject** | | | | | | | | |
| --- | --- | --- | --- | --- | --- | --- | --- | --- | --- |
| **ASV** | **#1** | **#2** | **#3** | **#4** | **#5** | **#6** | **#7** | **#8** | **#9** |
| 65f4 | + | + | + | + | + | + | + | + | + |
| a45d | Ns | Ns | - | + | - | + | + | - | Ns |
| 76b3 | - | + | - | Ns | - | - | Ns | - | Ns |
| f8d4 | + | Ns | - | - | - | - | + | Ns | Ns |
| ace8 | - | - | - | - | - | - | - | - | + |
| c588 | - | Ns | - | Ns | Ns |  | - | - | + |

+ p < 0.05

- not present

Ns p > 0.05

p-values calculated with STAMP (27).
