# Supplementary material for: Gut-derived Flavonifractor species variants are differentially enriched during in vitro incubation with quercetin
Source: PLoS One. 2020 Dec 2;15(12):e0227724. doi: 10.1371/journal.pone.0227724 (PMC7710108; doi:10.1371/journal.pone.0227724)
Supplement: S5 Table — (DOCX) [file pone.0227724.s012.docx]

**S5 Table.** **Estimates of Evolutionary Divergence between Amplicon Sequence Variants (ASVs) and Reference sequences.** The number of base substitutions per site from between sequences are shown. Analyses were conducted using the Maximum Composite Likelihood model (30). The analysis involved 44 nucleotide sequences (for simplicity only 25 organisms are shown). All positions containing gaps and missing data were eliminated. There were a total of 225 positions in the final dataset. Evolutionary analyses were conducted in MEGA6 (29).

| Organism or ASV | 1 | 2 | 3 | 4 | 5 | 6 | 7 | 8 | 9 | 10 | 11 | 12 | 13 | 14 | 15 | 16 | 17 | 18 | 19 | 20 | 21 | 22 | 23 | 24 | 25 |
| --- | --- | --- | --- | --- | --- | --- | --- | --- | --- | --- | --- | --- | --- | --- | --- | --- | --- | --- | --- | --- | --- | --- | --- | --- | --- |
| 1. ASV_65f4 |  |  |  |  |  |  |  |  |  |  |  |  |  |  |  |  |  |  |  |  |  |  |  |  |  |
| 2. ASV_a45d | 0.04 |  |  |  |  |  |  |  |  |  |  |  |  |  |  |  |  |  |  |  |  |  |  |  |  |
| 3. ASV_76b3 | 0.06 | 0.05 |  |  |  |  |  |  |  |  |  |  |  |  |  |  |  |  |  |  |  |  |  |  |  |
| 4. ASV_f8d4 | 0.07 | 0.06 | 0.03 |  |  |  |  |  |  |  |  |  |  |  |  |  |  |  |  |  |  |  |  |  |  |
| 5. ASV_ace8 | 0.16 | 0.18 | 0.17 | 0.18 |  |  |  |  |  |  |  |  |  |  |  |  |  |  |  |  |  |  |  |  |  |
| 6. ASV_c588 | 0.16 | 0.17 | 0.17 | 0.17 | 0.00 |  |  |  |  |  |  |  |  |  |  |  |  |  |  |  |  |  |  |  |  |
| 7. *F. plautii*_Y18187 | **0.00** | 0.04 | 0.06 | 0.07 | 0.16 | 0.16 |  |  |  |  |  |  |  |  |  |  |  |  |  |  |  |  |  |  |  |
| 8. *F. plauti*i_EU874848 | **0.00** | 0.04 | 0.06 | 0.07 | 0.16 | 0.16 | 0.00 |  |  |  |  |  |  |  |  |  |  |  |  |  |  |  |  |  |  |
| 9. *F. plauti*i_AY730662 | **0.00** | 0.04 | 0.06 | 0.07 | 0.16 | 0.16 | 0.00 | 0.00 |  |  |  |  |  |  |  |  |  |  |  |  |  |  |  |  |  |
| 10. *Flavonifractor*_AWSS01000025 | **0.00** | 0.04 | 0.06 | 0.07 | 0.16 | 0.16 | 0.00 | 0.00 | 0.00 |  |  |  |  |  |  |  |  |  |  |  |  |  |  |  |  |
| 11. *F. plautii*_AGCK01000014 | **0.00** | 0.04 | 0.06 | 0.07 | 0.16 | 0.16 | 0.00 | 0.00 | 0.00 | 0.00 |  |  |  |  |  |  |  |  |  |  |  |  |  |  |  |
| 12. *Flavonifractor* sp. An82 | 0.03 | **0.01** | 0.04 | 0.05 | 0.17 | 0.17 | 0.03 | 0.03 | 0.03 | 0.03 | 0.03 |  |  |  |  |  |  |  |  |  |  |  |  |  |  |
| 13. *Flavonifractor* sp. An306 | 0.03 | 0.02 | 0.05 | 0.06 | 0.18 | 0.18 | 0.03 | 0.03 | 0.03 | 0.03 | 0.03 | 0.02 |  |  |  |  |  |  |  |  |  |  |  |  |  |
| 14. *Flavonifractor* sp. An4 | 0.03 | **0.01** | 0.04 | 0.05 | 0.17 | 0.17 | 0.03 | 0.03 | 0.03 | 0.03 | 0.03 | 0.00 | 0.02 |  |  |  |  |  |  |  |  |  |  |  |  |
| 15. *P. capillosu*s ATCC 29799 | 0.04 | 0.04 | 0.07 | 0.08 | 0.17 | 0.17 | 0.04 | 0.04 | 0.04 | 0.04 | 0.04 | 0.04 | 0.05 | 0.04 |  |  |  |  |  |  |  |  |  |  |  |
| 16. *Intestinimonas*_FMGM01000011 | 0.07 | 0.06 | 0.03 | **0.00** | 0.18 | 0.17 | 0.07 | 0.07 | 0.07 | 0.07 | 0.07 | 0.05 | 0.06 | 0.05 | 0.08 |  |  |  |  |  |  |  |  |  |  |
| 17. *Intestinimonas*_HE974967 | 0.07 | 0.06 | 0.03 | **0.00** | 0.18 | 0.17 | 0.07 | 0.07 | 0.07 | 0.07 | 0.07 | 0.05 | 0.06 | 0.05 | 0.08 | 0.00 |  |  |  |  |  |  |  |  |  |
| 18. *Intestinimonas*_KY285278 | 0.07 | 0.06 | **0.01** | 0.02 | 0.18 | 0.18 | 0.07 | 0.07 | 0.07 | 0.07 | 0.07 | 0.05 | 0.06 | 0.05 | 0.08 | 0.02 | 0.02 |  |  |  |  |  |  |  |  |
| 19. *I. butyriciproducens*_MJII01000001.3039866 | 0.07 | 0.06 | 0.03 | **0.00** | 0.18 | 0.17 | 0.07 | 0.07 | 0.07 | 0.07 | 0.07 | 0.05 | 0.06 | 0.05 | 0.08 | 0.00 | 0.00 | 0.02 |  |  |  |  |  |  |  |
| 20. *I. butyriciproducens*_KC311367 | 0.07 | 0.06 | 0.03 | **0.00** | 0.18 | 0.17 | 0.07 | 0.07 | 0.07 | 0.07 | 0.07 | 0.05 | 0.06 | 0.05 | 0.08 | 0.00 | 0.00 | 0.02 | 0.00 |  |  |  |  |  |  |
| 21. *I. timonensis*_LN870298 | 0.07 | 0.06 | 0.03 | 0.03 | 0.19 | 0.18 | 0.07 | 0.07 | 0.07 | 0.07 | 0.07 | 0.05 | 0.06 | 0.05 | 0.08 | 0.03 | 0.03 | 0.03 | 0.03 | 0.03 |  |  |  |  |  |
| 22. *E. ramulus*_CYYA01000009 | 0.16 | 0.17 | 0.17 | 0.17 | **0.00** | **0.00** | 0.16 | 0.16 | 0.16 | 0.16 | 0.16 | 0.17 | 0.18 | 0.17 | 0.17 | 0.17 | 0.17 | 0.18 | 0.17 | 0.17 | 0.18 |  |  |  |  |
| 23. *E. ramulus*_AJ011522 | 0.16 | 0.17 | 0.17 | 0.17 | **0.00** | **0.00** | 0.16 | 0.16 | 0.16 | 0.16 | 0.16 | 0.17 | 0.18 | 0.17 | 0.17 | 0.17 | 0.17 | 0.18 | 0.17 | 0.17 | 0.18 | 0.00 |  |  |  |
| 24. *E. ramulus*_CYYA01000009(2) | 0.16 | 0.17 | 0.17 | 0.17 | **0.00** | **0.00** | 0.16 | 0.16 | 0.16 | 0.16 | 0.16 | 0.17 | 0.18 | 0.17 | 0.17 | 0.17 | 0.17 | 0.18 | 0.17 | 0.17 | 0.18 | 0.00 | 0.00 |  |  |
| 25. *E. ramulu*s_LG085505 | 0.16 | 0.17 | 0.17 | 0.17 | **0.00** | **0.00** | 0.16 | 0.16 | 0.16 | 0.16 | 0.16 | 0.17 | 0.18 | 0.17 | 0.17 | 0.17 | 0.17 | 0.18 | 0.17 | 0.17 | 0.18 | 0.00 | 0.00 | 0.00 |  |
| 26. *E. ramulus* ATCC 29099 | 0.16 | 0.17 | 0.17 | 0.17 | **0.00** | **0.00** | 0.16 | 0.16 | 0.16 | 0.16 | 0.16 | 0.17 | 0.18 | 0.17 | 0.17 | 0.17 | 0.17 | 0.18 | 0.17 | 0.17 | 0.18 | 0.00 | 0.00 | 0.00 | 0.00 |
