# Supplementary material for: Gut-derived Flavonifractor species variants are differentially enriched during in vitro incubation with quercetin
Source: PLoS One. 2020 Dec 2;15(12):e0227724. doi: 10.1371/journal.pone.0227724 (PMC7710108; doi:10.1371/journal.pone.0227724)
Supplement: S6 Table — (DOCX) [file pone.0227724.s013.docx]

**S6 Table**. **Protein list for orthologous gene clusters enriched in ASV_65f4-related genomes**. ASV_65f4-related group includes *F. plautii* YL31, *F. plautii* 2789STDY5834932, *F. plautii* ATCC 29863, *F. plautii* An248.

| **# cluster_name** | **protein_number** | **swiss_prot_id** | **go_annotation** | **protein_list** |
| --- | --- | --- | --- | --- |
| cluster2635 | 4 | Q892D0 | GO:0046336; P:ethanolamine catabolic process; IEA:UniProtKB-UniPathway | 2789STDY5834932\|CUP71372.1;An248\|OUO82145.1;YL31\|ANU41982.1;ATCC_29863\|EHM42371.1 |
| cluster2683 | 4 | Q9ZFV2 | GO:0046336; P:ethanolamine catabolic process; IEA:UniProtKB-UniPathway | 2789STDY5834932\|CUP71298.1;An248\|OUO82147.1;YL31\|ANU41984.1;ATCC_29863\|EHM42369.1 |
| cluster2711 | 4 | P0AEJ7 | GO:0046336; P:ethanolamine catabolic process; IEA:UniProtKB-UniPathway | 2789STDY5834932\|CUP71331.1;An248\|OUO82146.1;YL31\|ANU41983.1;ATCC_29863\|EHM42370.1 |
| cluster2744 | 4 | Q892D0 | GO:0046336; P:ethanolamine catabolic process; IEA:UniProtKB-UniPathway | 2789STDY5834932\|CUQ41078.1;An248\|OUO83463.1;YL31\|ANU42383.1;ATCC_29863\|EHM47953.1 |
| cluster2792 | 4 | P76552 | GO:0046336; P:ethanolamine catabolic process; IEA:UniProtKB-UniPathway | 2789STDY5834932\|CUP71884.1;An248\|OUO82132.1;YL31\|ANU41970.1;ATCC_29863\|EHM42337.1 |
| cluster2801 | 4 | P77277 | GO:0046336; P:ethanolamine catabolic process; IEA:UniProtKB-UniPathway | 2789STDY5834932\|CUP71680.1;An248\|OUO82137.1;YL31\|ANU41975.1;ATCC_29863\|EHM42332.1 |
| cluster2809 | 4 | Q9ZFV4 | GO:0046336; P:ethanolamine catabolic process; IEA:UniProtKB-UniPathway | 2789STDY5834932\|CUP71597.1;An248\|OUO82139.1;YL31\|ANU41977.1;ATCC_29863\|EHM42330.1 |
| cluster2881 | 4 | P76541 | GO:0046336; P:ethanolamine catabolic process; IEA:UniProtKB-UniPathway | 2789STDY5834932\|CUP71414.1;An248\|OUO82144.1;YL31\|ANU41981.1;ATCC_29863\|EHM42372.1 |
| cluster2965 | 4 | P0ABF5 | GO:0046336; P:ethanolamine catabolic process; IEA:UniProtKB-UniPathway | 2789STDY5834932\|CUP71451.1;An248\|OUO82143.1;YL31\|ANU41980.1;ATCC_29863\|EHM42373.1 |
| cluster3013 | 4 | P76552 | GO:0046336; P:ethanolamine catabolic process; IEA:UniProtKB-UniPathway | 2789STDY5834932\|CUP57828.1;An248\|OUO83125.1;YL31\|ANU42625.1;ATCC_29863\|EHM49955.1 |
| cluster3062 | 4 | P19264 | GO:0046336; P:ethanolamine catabolic process; IBA:GO_Central | 2789STDY5834932\|CUQ41065.1;An248\|OUO83464.1;YL31\|ANU42382.1;ATCC_29863\|EHM47952.1 |
| cluster2633 | 4 | E3PY99 | GO:0006591; P:ornithine metabolic process; TAS:UniProtKB | 2789STDY5834932\|CUQ29162.1;An248\|OUO83539.1;YL31\|ANU42311.1;ATCC_29863\|EHM54568.1 |
| cluster2723 | 4 | C1FW08 | GO:0006591; P:ornithine metabolic process; IDA:UniProtKB | 2789STDY5834932\|CUQ29280.1;An248\|OUO83532.1;YL31\|ANU42318.1;ATCC_29863\|EHM54561.1 |
| cluster2827 | 4 | E3PY97 | GO:0006591; P:ornithine metabolic process; IDA:UniProtKB | 2789STDY5834932\|CUQ29196.1;An248\|OUO83537.1;YL31\|ANU42313.1;ATCC_29863\|EHM54566.1 |
| cluster2982 | 4 | E3PY98 | GO:0006591; P:ornithine metabolic process; IDA:UniProtKB | 2789STDY5834932\|CUQ29181.1;An248\|OUO83538.1;YL31\|ANU42312.1;ATCC_29863\|EHM54567.1 |
| cluster2731 | 4 | P0A1D2 | GO:0031469; C:polyhedral organelle; IEA:InterPro | 2789STDY5834932\|CUP71147.1;An248\|OUO82151.1;YL31\|ANU41988.1;ATCC_29863\|EHM42365.1 |
| cluster2871 | 4 | P0A1D2 | GO:0031469; C:polyhedral organelle; IEA:InterPro | 2789STDY5834932\|CUP99707.1;An248\|OUO83195.1;YL31\|ANU42686.1;ATCC_29863\|EHM52290.1 |
| cluster2952 | 4 | P0A1D2 | GO:0031469; C:polyhedral organelle; IEA:InterPro | 2789STDY5834932\|CUP99735.1;An248\|OUO83196.1;YL31\|ANU42687.1;ATCC_29863\|EHM52288.1 |
| cluster2244 | 5 | P23446 | GO:0071978; P:bacterial-type flagellum-dependent swarming motility; IBA:GO_Central | 2789STDY5834932\|CUP86983.1;An248\|OUO84117.1;YL31\|ANU40129.1;An306\|OUO41293.1;ATCC_29863\|EHM53550.1 |
| cluster2270 | 5 | P39063 | GO:0071978; P:bacterial-type flagellum-dependent swarming motility; IBA:GO_Central | 2789STDY5834932\|CUP87040.1;An248\|OUO84119.1;YL31\|ANU40131.1;An306\|OUO41291.1;ATCC_29863\|EHM53552.1 |
| cluster2305 | 5 | P24501 | GO:0071978; P:bacterial-type flagellum-dependent swarming motility; IBA:GO_Central | 2789STDY5834932\|CUP86639.1;An248\|OUO84107.1;YL31\|ANU40119.2;An306\|OUO41301.1;ATCC_29863\|EHM53539.1 |
| cluster2336 | 5 | P23446 | GO:0071978; P:bacterial-type flagellum-dependent swarming motility; IBA:GO_Central | 2789STDY5834932\|CUP87427.1;An248\|OUO84131.1;YL31\|ANU40143.1;An306\|OUO41279.1;ATCC_29863\|EHM53564.1 |
| cluster2463 | 5 | P24500 | GO:0071978; P:bacterial-type flagellum-dependent swarming motility; IBA:GO_Central | 2789STDY5834932\|CUP86599.1;An248\|OUO84106.1;YL31\|ANU40118.1;An306\|OUO41302.1;ATCC_29863\|EHM53538.1 |
| cluster2194 | 5 | Q0AXB7 | GO:0006935; P:chemotaxis; IEA:UniProtKB-UniRule | 2789STDY5834932\|CUP86231.1;An248\|OUO84097.1;YL31\|ANU40110.1;An306\|OUO31958.1;ATCC_29863\|EHM38114.1 |
| cluster2239 | 5 | Q9X006 | GO:0006935; P:chemotaxis; IEA:UniProtKB-KW | 2789STDY5834932\|CUP87493.1;An248\|OUO84133.1;YL31\|ANU40145.1;An306\|OUO41277.1;ATCC_29863\|EHM53566.1 |
| cluster2240 | 5 | P21813 | GO:0006935; P:chemotaxis; IEA:UniProtKB-KW | 2789STDY5834932\|CUP86340.1;An248\|OUO84100.1;YL31\|ANU40113.1;An306\|OUO31960.1;ATCC_29863\|EHM38117.1 |
| cluster2319 | 5 | P23453 | GO:0050918; P:positive chemotaxis; IMP:CACAO | 2789STDY5834932\|CUP87107.1;An248\|OUO84121.1;YL31\|ANU40133.2;An306\|OUO41289.1;ATCC_29863\|EHM53554.1 |
| cluster2350 | 5 | Q0AYK9 | GO:0006935; P:chemotaxis; IEA:UniProtKB-UniRule | 2789STDY5834932\|CUP87524.1;An248\|OUO84134.1;YL31\|ANU40146.1;An306\|OUO41276.1;ATCC_29863\|EHM53567.1 |
| cluster2437 | 5 | Q9WY63 | GO:0006935; P:chemotaxis; IEA:UniProtKB-KW | 2789STDY5834932\|CUP86742.1;An248\|OUO84110.1;YL31\|ANU40122.1;An306\|OUO41299.1;ATCC_29863\|EHM53542.1 |
| cluster2210 | 5 | O67750 | GO:0009306; P:protein secretion; IEA:InterPro | 2789STDY5834932\|CUP87233.1;An248\|OUO84125.1;YL31\|ANU40137.1;An306\|OUO41285.1;ATCC_29863\|EHM53558.1 |
| cluster2343 | 5 | P35620 | GO:0009306; P:protein secretion; IEA:InterPro | 2789STDY5834932\|CUP87365.1;An248\|OUO84129.1;YL31\|ANU40141.1;An306\|OUO41281.1;ATCC_29863\|EHM53562.1 |
| cluster2363 | 5 | P35535 | GO:0009306; P:protein secretion; IEA:InterPro | 2789STDY5834932\|CUP87266.1;An248\|OUO84126.1;YL31\|ANU40138.1;An306\|OUO41284.1;ATCC_29863\|EHM53559.1 |
| cluster2453 | 5 | P35538 | GO:0009306; P:protein secretion; IEA:InterPro | 2789STDY5834932\|CUP87336.1;An248\|OUO84128.1;YL31\|ANU40140.1;An306\|OUO41282.1;ATCC_29863\|EHM53561.1 |
| cluster1763 | 7 | P80583 | GO:0071973; P:bacterial-type flagellum-dependent cell motility; IEA:InterPro | 2789STDY5834932\|CUP86109.1;An248\|OUO84093.1;YL31\|ANU40106.1;An306\|OUO41062.1;ATCC_29863\|EHM38107.1;An306\|OUO39651.1;An306\|OUO31954.1 |
| cluster2130 | 5 | P39810 | GO:0071973; P:bacterial-type flagellum-dependent cell motility; IEA:InterPro | 2789STDY5834932\|CUQ42182.1;An248\|OUO85163.1;YL31\|ANU40099.1;An306\|OUO42493.1;ATCC_29863\|EHM43411.1 |
| cluster2207 | 5 | A1SEQ0 | GO:0071973; P:bacterial-type flagellum-dependent cell motility; IEA:InterPro | 2789STDY5834932\|CUP86675.1;An248\|OUO84108.1;YL31\|ANU40120.1;An306\|OUO41312.1;ATCC_29863\|EHM53540.1 |
| cluster2220 | 5 | P24073 | GO:1902021; P:regulation of bacterial-type flagellum-dependent cell motility; IMP:CACAO | 2789STDY5834932\|CUP87135.1;An248\|OUO84122.1;YL31\|ANU40134.1;An306\|OUO41288.1;ATCC_29863\|EHM53555.1 |
| cluster2486 | 5 | Q8K9K4 | GO:0071973; P:bacterial-type flagellum-dependent cell motility; IEA:InterPro | 2789STDY5834932\|CUP87460.1;An248\|OUO84132.1;YL31\|ANU40144.1;An306\|OUO41278.1;ATCC_29863\|EHM53565.1 |
