# Supplementary material for: Gut-derived Flavonifractor species variants are differentially enriched during in vitro incubation with quercetin
Source: PLoS One. 2020 Dec 2;15(12):e0227724. doi: 10.1371/journal.pone.0227724 (PMC7710108; doi:10.1371/journal.pone.0227724)
Supplement: S7 Table — (DOCX) [file pone.0227724.s014.docx]

**S7 Table.** **Protein list for orthologous gene clusters enriched in ASV_a45d-related genomes.** ASV_a45d-related group includes *Flavonifractor* sp. An4, *Flavonifractor* sp. An9, *Flavonifractor* sp. An82, *Flavonifractor* sp. An306.

| **# cluster_name** | **protein_number** | **swiss_prot_id** | **go_annotation** | **protein_list** |
| --- | --- | --- | --- | --- |
| cluster2102 | 5 | Q2RGL2 | GO:0006777; P:Mo-molybdopterin cofactor biosynthetic process; IEA:UniProtKB-UniRule | An82\|OUN22106.1;An4\|OUO17000.1;An10\|OUQ81968.1;An306\|OUO42827.1;An306\|OUO41792.1 |
| cluster2541 | 5 | P44902 | GO:0006777; P:Mo-molybdopterin cofactor biosynthetic process; IEA:UniProtKB-KW | An4\|OUO16999.1;An82\|OUN22107.1;An10\|OUQ81967.1;An306\|OUO42828.1;An306\|OUO41791.1 |
| cluster3168 | 4 | B6IQ15 | GO:0006777; P:Mo-molybdopterin cofactor biosynthetic process; IEA:UniProtKB-UniRule | An4\|OUO16998.1;An82\|OUN22108.1;An10\|OUQ81966.1;An306\|OUO42829.1 |
| cluster3210 | 4 | Q8YY90 | GO:0006777; P:Mo-molybdopterin cofactor biosynthetic process; IEA:UniProtKB-KW | An4\|OUO16450.1;An82\|OUN21730.1;An306\|OUO41795.1;An10\|OUQ83256.1 |
| cluster3198 | 4 | Q5WH08 | GO:0009231; P:riboflavin biosynthetic process; IEA:UniProtKB-UniRule | An4\|OUO17295.1;An82\|OUN22005.1;An10\|OUQ80892.1;An306\|OUO44622.1 |
| cluster3201 | 4 | B2V4J4 | GO:0009231; P:riboflavin biosynthetic process; IEA:UniProtKB-UniRule | An4\|OUO17294.1;An82\|OUN22004.1;An10\|OUQ80891.1;An306\|OUO44623.1 |
| cluster3206 | 4 | P50854 | GO:0009231; P:riboflavin biosynthetic process; IEA:UniProtKB-UniPathway | An4\|OUO17296.1;An82\|OUN22006.1;An10\|OUQ80893.1;An306\|OUO44621.1 |
| cluster3212 | 4 | Q97EZ4 | GO:0006012; P:galactose metabolic process; IEA:UniProtKB-UniRule | An4\|OUO16988.1;An82\|OUN20983.1;An10\|OUQ83371.1;An306\|OUO41913.1 |
| cluster3213 | 4 | A5VME2 | GO:0006012; P:galactose metabolic process; IEA:UniProtKB-UniRule | An4\|OUO16990.1;An82\|OUN20981.1;An10\|OUQ83373.1;An306\|OUO41915.1 |
