# Supplementary material for: Gut-derived Flavonifractor species variants are differentially enriched during in vitro incubation with quercetin
Source: PLoS One. 2020 Dec 2;15(12):e0227724. doi: 10.1371/journal.pone.0227724 (PMC7710108; doi:10.1371/journal.pone.0227724)
Supplement: S8 Table — (DOCX) [file pone.0227724.s015.docx]

**S8 Table. Ids for Eut and Pdu proteins for *Flavonifractor* spp.**

| **Protein** | **YL31** | **ATCC_29863** | **2789STDY5834932** | **An248** | **An306** | **An82** | **An10** | **An4** |
| --- | --- | --- | --- | --- | --- | --- | --- | --- |
| ***First Eut operon*** |  |  |  |  |  |  |  |  |
| **AlcDH** | ANU39943.1 | EHM41053.1 | CUP45427.1 | OUO85060.1 | OUO39463.1 | OUN22497.1 | OUQ83249.1 | OUO11851.1 |
| **EutA** | ANU39944.1 | EHM41052.1 | CUP45465.1 | OUO85061.1 | OUO39462.1 | OUN22498.1 | OUQ83248.1 | OUO11850.1 |
| **EutB** | ANU39945.1 | EHM41051.1 | CUP45499.1 | OUO85062.1 | OUO39461.1 | OUN22499.1 | OUQ83247.1 | OUO11849.1 |
| **EutC** | ANU39946.1 | EHM41050.1 | CUP45537.1 | OUO85063.1 | OUO39460.1 | OUN22500.1 | OUQ83246.1 | OUO11848.1 |
| **EutL** | ANU39947.1 | EHM41048.1 | CUP45573.1 | OUO85064.1 | OUO39459.1 | OUN22501.1 | OUQ83245.1 | OUO11846.1 |
| **AldDH** | ANU39948.1 | EHM41078.1 | CUP45615.1 | OUO85065.1 | OUO39458.1 | OUN22502.1 | OUQ83244.1 | OUO11845.1 |
| **EutM** | CP015406.2: 490,436-491,117 | EHM41077.1 | CUP45688.1 | NFJM01000001.1:255050-255725 | OUO39492.1 | OUN22646.1 | OUQ83425.1 | OUO11857.1 |
| **EutT** | ANU39949.1 | EHM41076.1 | CUP45722.1 | OUO85066.1 | OUO39457.1 | OUN22503.1 | OUQ83243.1 | OUO11844.1 |
| **PTAC** | ANU39950.1 | EHM41075.1 | CUP45761.1 | OUO85067.1 | OUO39456.1 | OUN22504.1 | OUQ83242.1 | OUO11843.1 |
| **36% identity with ethanolamine utilization protein** | ANU39951.1 | EHM41074.1 | CUP45794.1 | OUO85068.1 | OUO39455.1 | OUN22505.1 | OUQ83241.1 | OUO11842.1 |
| **EutN** | ANU39952.1 | EHM41073.1 | CUP45828.1 | OUO85069.1 | OUO39454.1 | OUN22506.1 | OUQ83240.1 | OUO11841.1 |
| **EutH** | ANU39953.1 | EHM41072.1 | CUP45859.1 | OUO85070.1 | OUO39453.1 | OUN22507.1 | OUQ83239.1 | OUO11840.1 |
| **EutQ** | ANU39954.1 | EHM41071.1 | CUP45901.1 | OUO85071.1 | OUO39452.1 | OUN22508.1 | OUQ83238.1 | OUO11839.1 |
|  |  |  |  |  |  |  |  |  |
| ***Pdu Operon*** |  |  |  |  |  |  |  |  |
| **PduV** | ANU41514.1 | EHM40047.1 | CUP30877.1 | OUO82753.1 | OUO37647.1 | OUN20352.1 |  |  |
| **PduU** | ANU41515.2 | EHM40046.1 | CUP30904.1 | OUO82944.1 | OUO37656.1 | OUN20407.1 |  |  |
| **oxidoreductase** | ANU41516.1 | EHM40044.1 | CUP30965.1 | OUO82752.1 | OUO37645.1 | OUN20350.1 |  |  |
| **PduT** | ANU41517.1 | EHM40043.1 | CUP30990.1 | OUO82751.1 | OUO37644.1 | OUN20349.1 |  |  |
| **hypothetical protein** | ANU41518.1 | EHM40042.1 | CUP31020.1 | OUO82750.1 | OUO37655.1 | OUN20348.1 |  |  |
| **PduS** | ANU41519.1 | EHM40041.1 | CUP31053.1 | OUO82749.1 | OUO37643.1 | OUN20347.1 |  |  |
| **AldDH** | ANU41520.1 | EHM40040.1 | CUP31081.1 | OUO82748.1 | OUO37642.1 | OUN20346.1 |  |  |
| **PduO** | ANU41521.1 | EHM40039.1 | CUP31110.1 | OUO82747.1 | OUO37641.1 | OUN20345.1 |  |  |
| **PduN** | ANU41522.1 | EHM40038.1 | CUP31136.1 | OUO82746.1 | OUO37640.1 | OUN20344.1 |  |  |
| **PduM** | ANU41523.1 | EHM40037.1 | CUP31164.1 | OUO82745.1 | OUO37639.1 | OUN20343.1 |  |  |
| **PTAC** | ANU41524.1 | EHM40035.1 | CUP31220.1 | OUO82743.1 | OUO37637.1 | OUN20341.1 |  |  |
| **PduA** | ANU41525.1 | EHM40034.1 | CUP31258.1 | OUO82742.1 | OUO37636.1 | OUN20340.1 |  |  |
| **PduJ** | ANU41526.1 | EHM40033.1 | CUP31293.1 | OUO82741.1 | OUO37635.1 | OUN20339.1 (contig break) |  | |
| **PduH** | ANU41527.1 | EHM40032.1 | CUP31326.1 | OUO82740.1 | OUO37634.1 | OUN18837.1 |  |  |
| **PduG** | ANU41528.1 | EHM40031.1 | CUP31355.1 | OUO82739.1 | OUO37633.1 | OUN18838.1 |  |  |
| **PduE** | ANU41529.1 | EHM40030.1 | CUP31392.1 | OUO82738.1 | OUO37632.1 | OUN18839.1 |  |  |
| **PduD** | ANU41530.1 | EHM40029.1 | CUP31436.1 | OUO82737.1 | OUO37631.1 | OUN18840.1 |  |  |
| **PduC** | ANU41531.1 | EHM40028.1 | CUP31491.1 | OUO82736.1 | OUO37630.1 | OUN18841.1 |  |  |
| **PduB** | ANU41532.1 | EHM40027.1 | CUP31544.1 | OUO82735.1 | OUO37629.1 | OUN18842.1 |  |  |
| **PduJ** | ANU41533.1 | EHM40026.1 | CUP31585.1 | OUO82734.1 | OUO37628.1 | OUN18843.1 |  |  |
| **AlcDH** | ANU41534.1 | EHM40025.1 | CUP31631.1 | OUO82733.1 | OUO37626.1 | OUN18844.1 |  |  |
| **Regulator** | ANU41535.1 | EHM40024.1 | CUP31669.1 | OUO82732.1 | OUO37625.1 | OUN18845.1 |  |  |
| **Histidine kinase** | ANU41536.1 | EHM40023.1 | CUP31727.1 | OUO82731.1 | OUO37624.1 | OUN18846.1 |  |  |
| **Kinase** | ANU41537.1 | EHM40021.1 | CUP31777.1 | OUO82730.1 | contig break | contig break |  |  |
|  |  |  |  |  |  |  |  |  |
| ***Second Eut operon*** |  |  |  |  |  |  |  |  |
| **EutQ** | ANU41969.1 | EHM42338.1 | CUP71918.1 | OUO82131.1 |  |  |  |  |
| **EutH** | ANU41970.1 | EHM42337.1 | CUP71884.1 | OUO82132.1 |  |  |  |  |
| **BMC protein** | ANU41971.1 | EHM42336.1 | CUP71851.1 | OUO82133.1 |  |  |  |  |
| **PduS homolog, cobalamin reductase** | ANU41972.1 | EHM42335.1 | CUP71805.1 | OUO82134.1 |  |  |  |  |
| **EutN** | ANU41973.1 | EHM42334.1 | CUP71761.1 | OUO82135.1 |  |  |  |  |
| **36% identity with PduM** | ANU41974.1 | EHM42333.1 | CUP71724.1 | OUO82136.1 |  |  |  |  |
| **EutJ** | ANU41975.1 | EHM42332.1 | CUP71680.1 | OUO82137.1 |  |  |  |  |
| **PTAC** | ANU41976.1 | EHM42331.1 | CUP71633.1 | OUO82138.1 |  |  |  |  |
| **EutT** | ANU41977.1 | EHM42330.1 | CUP71597.1 | OUO82139.1 |  |  |  |  |
| **EutM** | ANU41978.1 | EHM42329.1 | CUP71551.1 | OUO82140.1 |  |  |  |  |
| **EutM** | ANU41979.1 | EHM42328.1 | CUP71511.1 | OUO82141.1 |  |  |  |  |
| **EutM** | ANU41980.1 | EHM42373.1 | CUP71451.1 | OUO82143.1 |  |  |  |  |
| **EutL** | ANU41981.1 | EHM42372.1 | CUP71414.1 | OUO82144.1 |  |  |  |  |
| **EutC** | ANU41982.1 | EHM42371.1 | CUP71372.1 | OUO82145.1 |  |  |  |  |
| **EutB** | ANU41983.1 | EHM42370.1 | CUP71331.1 | OUO82146.1 |  |  |  |  |
| **EutA** | ANU41984.1 | EHM42369.1 | CUP71298.1 | OUO82147.1 |  |  |  |  |
| **pdtaS; two-component system, sensor histidine kinase PdtaS [EC:2.7.13.3]** | ANU41985.1 | EHM42368.1 | CUP71252.1 | OUO82148.1 |  |  |  |  |
| **pdtaR; two-component system, response regulator PdtaR** | ANU41986.1 | EHM42367.1 | CUP71219.1 | OUO82149.1 |  |  |  |  |
| **EutP** | ANU41987.1 | EHM42366.1 | CUP71185.1 | OUO82150.1 |  |  |  |  |
| **EutS** | ANU41988.1 | EHM42365.1 | CUP71147.1 | OUO82151.1 |  |  |  |  |
| **AlcDH/AldDH** | ANU41989.1 | EHM42364.1 | CUP71114.1 | OUO82152.1 |  |  |  |  |
