# Supplementary material for: Gut-derived Flavonifractor species variants are differentially enriched during in vitro incubation with quercetin
Source: PLoS One. 2020 Dec 2;15(12):e0227724. doi: 10.1371/journal.pone.0227724 (PMC7710108; doi:10.1371/journal.pone.0227724)
Supplement: S9 Table — (DOCX) [file pone.0227724.s016.docx]

**S9 Table**. **Distance Matrix for Pdu Operon in *F. plautii* YL31 with the one in *Salmonella enterica* subsp. *enterica* serovar Typhimurium str. LT2.**

| ***F. plautii* YL31** | ***Salmonella enterica*** | **Distance^a^** |
| --- | --- | --- |
| ANU41514.1 | NP 461001.1 PduV | 1.124 |
| ANU41515.2 | NP 461000.1 PduU | 0.431 |
| ANU41517.1 | NP 460999.1 PduT | 0.856 |
| ANU41519.1 | NP 460998.1 PduS | 0.856 |
| ANU41520.1 | NP 460996.1 PduP | 0.431 |
| ANU41521.1 | NP 460995.1 PduO | 0.744 |
| ANU41522.1 | NP 460994.1 PduN | 0.744 |
| ANU41524.1 | NP 460992.1 PduL | 0.470 |
| ANU41525.1 | NP 460983.1 PduA | 0.255 |
| ANU41526.1 | NP 460990.1 PduJ | 0.393 |
| ANU41527.1 | NP 460989.1 PduH | 0.916 |
| ANU41528.1 | NP 460988.1 PduG | 0.693 |
| ANU41529.1 | NP 460987.1 PduE | 0.322 |
| ANU41530.1 | NP 460986.1 PduD | 0.431 |
| ANU41531.1 | NP 460985.1 PduC | 0.105 |
| ANU41532.1 | NP 460984.3 PduB | 0.357 |
| ANU41533.1 | NP 460990.1 PduJ | 0.223 |
| ANU41534.1 | NP 460997.1 PduQ | 0.393 |

^a^Estimates of Evolutionary Divergence between Sequences, the number of amino acid substitutions per site from between sequences are shown. Analyses were conducted using the Poisson correction model (70). The analysis involved 42 amino acid sequences. All positions containing gaps and missing data were eliminated. There were a total of 40 positions in the final dataset. Evolutionary analyses were conducted in MEGA6.
